# DCLK1 isoform (DCLK1-S) as a critical player in promoting inflammation, tissue remodeling, and EMT in mouse models of colitis

**DOI:** 10.1371/journal.ppat.1013360

**Published:** 2025-08-21

**Authors:** Kafayat Yusuf, Badal C Roy, William L Hauser, Mazin Al-Kasspooles, Sierra Sanchez, Thomas M Attard, Dong Pei, Venkatesh Sampath, Shrikant Anant, Shahid Umar

**Affiliations:** 1 Department of Surgery, University of Kansas Medical Center, Kansas City, Kansas, United States of America; 2 Department of Cancer Biology, University of Kansas Medical Center, Kansas City, Kansas, United States of America; 3 Department of Anesthesiology, University of Kansas Medical Center, Kansas City, Kansas, United States of America; 4 Department of Pediatric Gastroenterology, Children’s Mercy Hospital, Kansas City, Missouri, United States of America; 5 Department of Biostatistics and Data Science, University of Kansas Medical Center, Kansas City, Kansas, United States of America; 6 Department of Pediatrics/Neonatology, Children’s Mercy Hospital, Kansas City, Missouri, United States of America; University of Oklahoma Health Sciences Center, UNITED STATES OF AMERICA

## Abstract

**Background and Aims:**

The Doublecortin-like kinase-1 (DCLK1) plays a chemosensory role in the gut. It’s role in the context of inflammatory diseases including inflammatory bowel disease (IBD), has not been thoroughly investigated. This study explored the role of the DCLK1 isoform (DCLK1-S) in promoting infectious/chemical colitis by utilizing high-throughput imaging mass cytometry (IMC).

**Methods:**

Transgenic mice were either infected with Citrobacter rodentium (CR) or received DSS and tissues/cells were processed via standard techniques. IMC workflow was adapted by Fluidigm (renamed Standard BioTools). Raw data was fed to Multiplexed Cell Dataset (MCD) Viewer for image generation and analyzed via histoCAT. Promoters for DCLK1 long (DCLK1-L) and short (DCLK1-S) transcripts were cloned, and promoter activities were determined via luciferase reporter assays.

**Results:**

Following CR-induced infectious colitis in mice, IMC revealed accumulation of DCLK1-S in the colons of infected mice that inversely correlated with DCLK1-S repressor FoxD3 (Forkhead Box D3). Elevated DCLK1-S levels corresponded with MMP13 staining and activity, promoting collagen degradation and fibrosis. We confirmed the DCLK1-S/MMP13 axis in a knock- in mouse model overexpressing DCLK1-S, in conjunction with dextran sulfate sodium (DSS)- induced colitis. During DCLK1-L and DCLK1-S promoter-reporter assays, we observed a more dramatic decrease in DCLK1-S reporter activity in response to either MMP13 inhibitor, WAY- 170523 or DCLK1 inhibitor, DCLK1-IN-1 compared to the effect of these inhibitors on DCLK1-L promoter. Furthermore, we identified epithelial-to-mesenchymal transition (EMT) as a prelude to colitis.

**Conclusions:**

Persistent expression of DCLK1-S drives a severe inflammatory phenotype, contributing to extracellular matrix (ECM) remodeling, fibrosis, and EMT, thus playing pivotal roles in colitis pathogenesis and presenting potential avenues for novel treatment strategies.

## Author summary

Inflammatory Bowel Disease (IBD) is a chronic, relapsing inflammatory condition of the gastrointestinal tract that significantly affects patient quality of life. Despite available therapies, IBD remains incurable, and its global incidence is steadily increasing. To develop more effective treatments, it is crucial to understand the molecular drivers of chronic intestinal inflammation. In this study, we employed multiple murine models of colitis, including genetically modified mice, to explore disease mechanisms. These experimental models mimic key features of human Ulcerative Colitis and allow controlled investigation of specific molecular pathways involved in inflammation and tissue remodeling. Our findings demonstrate that overexpression of an isoform of DCLK1 drives a severe inflammatory phenotype, contributing to extracellular matrix (ECM) remodeling, fibrosis, and epithelial-to-mesenchymal transition (EMT). These effects were consistent across different colitis models, underscoring the pathogenic role of DCLK1-S in promoting chronic intestinal injury. Overall, this work highlights DCLK1-S as a promising isoform-specific target for therapeutic intervention in IBD and other inflammation-associated diseases.

## Introduction

Inflammatory bowel disease (IBD) is correlated with Ulcerative colitis (UC) and Crohn’s disease (CD), both persistent inflammation-related conditions of the intestinal tract [1,2]. Environmental, genetic, and behavioral factors all impact the occurrence and prevalence of IBD statistics, which have been rising in different regions worldwide, with the highest rates observed in North America and Europe [3]. While CD is often regarded as more debilitating than UC, the global occurrence of UC is significantly greater [4,5].

*Citrobacter rodentium*(CR) that resembles human pathogens including enteropathogenic *E.coli* and enterohemorrhagic *E.coli*, and Dextran Sodium Sulphate (DSS) are infectious and chemical-induced models of colitis respectively and are both widely used to mimic IBD [6].

DCLK1 (Doublecortin-like kinase 1) is a versatile protein with diverse roles in the gut. It serves as a marker for tuft cells, contributing to intestinal homeostasis [7–9]. Additionally, DCLK1 functions as a cancer stem cell marker in the colon [10–13]. Despite their diverse roles, few studies have examined the underlying mechanisms driving their alternative functions. O’Connell and colleagues were one of the first group of investigators to identify the two main isoforms of DCLK1 in the colon: DCLK1-L, the full-length protein, and DCLK1-S, a shorter variant lacking the two doublecortin DCX domains in its N-terminus [14]. The epigenetic repression of the $5^{\prime }$(*α*)-promoter of DCLK1 during early colon carcinogenesis leads to the loss of DCLK1-L expression and a subsequent DCLK1-S expression activated by an alternate-(*β*) promoter within IntronV of the DCLK1 gene [14]. Further research on isoform-specific functions of DCLK1 reveals distinct roles in disease pathogenesis. DCLK1-L, with its microtubule-binding doublecortin domains, acts as a marker for tuft cells, promoting homeostasis and epithelial regeneration [15–17]. In contrast, DCLK1-S is implicated in carcinogenesis, driving disease progression [18,19].

Intestinal fibrosis is common in people with IBD [20,21]. It signifies the culmination of the body’s response to ongoing inflammation, which triggers an extended wound-healing process, causing an excessive buildup of extracellular matrix (ECM). Over time, this accumulation of matrix leads to impaired intestinal function [22]. The process of epithelial-to-mesenchymal transition (EMT) mirrors a developmental program that is reactivated during wound healing, fibrosis, and cancer. Persistent inflammation in the context of IBD results in ongoing damage to the gut alongside fibrosis. Although the body initiates an inflammatory response as a natural wound-healing mechanism to restore intestinal integrity and repair damaged tissue, the resolution mechanisms are compromised when the injury persists. This event starts a vicious cycle in which persistent damage and attempted healing coexist, culminating in chronic inflammation. This persistent inflammation, coupled with the activation of EMT, contributes to the progression of fibrosis in the intestine [20].

Despite the crucial role of DCLK1 in gut function, its involvement in IBD remains relatively unexplored. This study aims to address this gap in knowledge by utilizing high-throughput imaging Mass Cytometry (IMC) to analyze the colon’s expression profile in mouse models that lack the DCLK1-L isoform in the colon. These new findings reveal a correlation between sustained expression of DCLK1-S, and inflammation, extracellular matrix degradation, and epithelial-to-mesenchymal transition (EMT), which are key indicators of IBD progression. Thus, we provide new insights into the critical role of DCLK1-S in the pathogenesis of infectious or DSS-induced colitis.

## Results

### Infection induces distinct compartmentalization of markers as revealed by imaging mass cytometry (IMC)

To establish the role of DCLK1 and its isoforms in the progression of colitis, we mated DCLK1^fl/fl^ mice with CDX2-Cre/ERT2 mice to develop DCLK1^ΔIEC^ mice using tamoxifen-induced Cre recombination as described previously [23]. There were three different groups of mice utilized for this study; In the control group, mice were administered with Tamoxifen to affect the epithelial deletion of DCLK1 (these are the uninfected controls), CR (mice were administered with tamoxifen and given oral CR infection to induce colitis), CR+DBZ (mice were administered tamoxifen, given oral CR infection and DBZ; Dibenzazepine, a *γ*-secretase inhibitor). The utilization of a Notch/*γ*-secretase inhibitor combined with CR infection often results in the disruption of the colon intestinal barrier and aggravation of colitis as previously described [23–25]. DBZ on its own, in the absence of CR infection, has no effect on colon biology [24]. S1A Fig illustrates a DNA overlay of tissue segments to visualize the spatial distribution of DNA inside the tissue samples. In this approach, the tissue segment is stained with a fluorescent dye that is specific to DNA, in this case, DAPI (4’,6-diamidino-2-phenylindole), which tethers to DNA and fluoresces when excited. The tissue segment is imaged for DAPI fluorescence to apply the DNA overlay after it has been labeled with different antibodies that target proteins or markers. DNA overlay staining is a useful technique for locating nuclei within tissue samples and establishing a correlation between DNA distribution and target protein expression patterns. This can provide valuable information about the cellular makeup and architecture of the tissue [26].

To allow for the interpretation of cellular behavior in its spatial context, we illustrate the cell overlay in S1B Fig to reveal and visualize the spatial distribution of several cell types within the tissue samples, this overlay allows for the interpretation of various cell populations within the tissue microenvironment [27].

To differentiate between the epithelial and stromal components of the tissue sections, we employed E-Cadherin and *α*-smooth muscle actin (*α*-SMA) staining (S1C and S1D Fig). Notably, there is discernible staining of E-cadherin-positive epithelial cells, distinctly separated from the *α*-SMA-positive populations within the tissues (S1C and S1D Fig). In the control group, an almost even distribution of epithelial and stromal cells is observed. However, this distribution pattern shifts in the CR and CR + DBZ groups, where the data indicates an increased expansion and intensity of E-cadherin-positive epithelial cells, concomitant with a reduction as colitis severity increases. Additionally, there is an increased expansion of *α*-SMA-positive cells in the CR group with non-distinct colocalization with E-cadherin in more severe colitis conditions (S1C and S1D Fig). S2A Fig provides details about the panel and antibody design. S2B and S2C Fig represent the phenograph overlay of cells showing distinct color staining and cell colocalization patterns in the three groups as well as tSNE distribution of discrete clusters. In S2D Fig, we found notable differences between the uninfected control groups, the CR group, and the CR+DBZ group. These findings unveil a distinct expression profile of epithelial and stromal cells in the colon under normal crypt architecture that transitions with heightened proliferative cells and a more distorted crypt architecture during inflammation.

### DCLK1-S persists during colon inflammation coinciding with Notch activity, and displaying an inverse correlation with FoxD3

**Fig 1:**
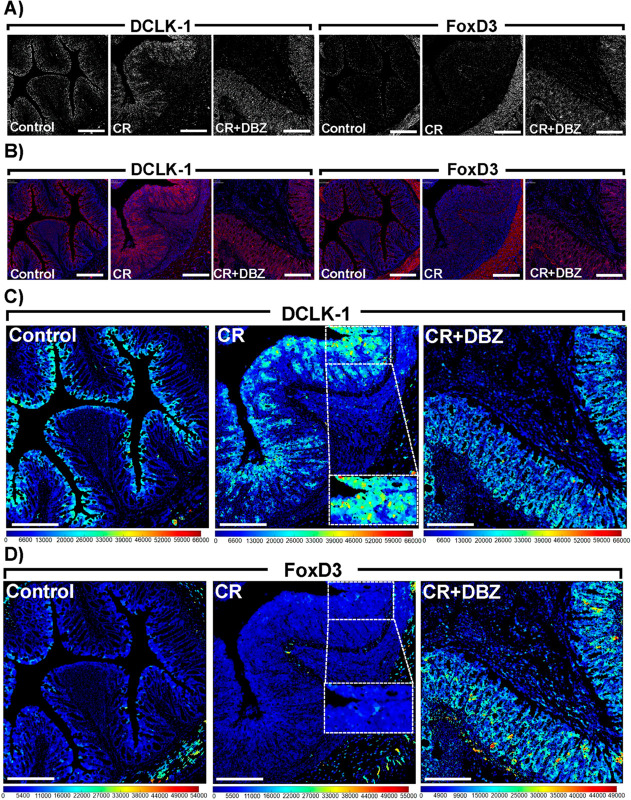
DCLK1 expression negatively correlated with FoxD3 status in the colons of DCLK1^ΔIEC^ mice. A, B. Bright-field and immunofluorescence (IMF) images of Control, CR or CR+DBZ-treated mouse colon sections showing DCLK1 and FoxD3 staining. C, D. Representative heatmap channels generated for DCLK1 and FoxD3 in the indicated groups using MCD-Viewer. Boxed areas in C and D represent magnified images of DCLK1 and FoxD3 captured at the same location to highlight differences in staining. Scale bar = 200 *μ*m; n = 2 independent experiments.

We systematically evaluated the effect of epithelial deletion of DCLK1-L in DCLK1^ΔIEC^ mice upon bacterial infection and inhibition of Notch pathway. In response to CR infection, increased DCLK1 expression was particularly noticeable with a decline in the CR+DBZ group (Fig 1A-C). To determine whether this expression pertained to the full-length DCLK1 (DCLK1-L) or the short form (DCLK1-S), we examined the expression status of Forkhead Box D3 (FoxD3), a protein known to regulate the short form of DCLK1 [28]. The staining pattern in the subsequent part of Fig 1A and 1B, and in Fig 1D, revealed a complete loss of FoxD3 expression, especially in regions with elevated DCLK1 expression. In Fig 1C, there was an increased intensity of DCLK1 expression in the crypt epithelial compartment of the colon in the CR group. Although this intensity slightly decreased in the CR+DBZ group (Fig 1C), it remained more abundant than in the uninfected control group. Conversely, there was a lack of FoxD3 expression when the same regions were analyzed (Fig 1D).

**Fig 2:**
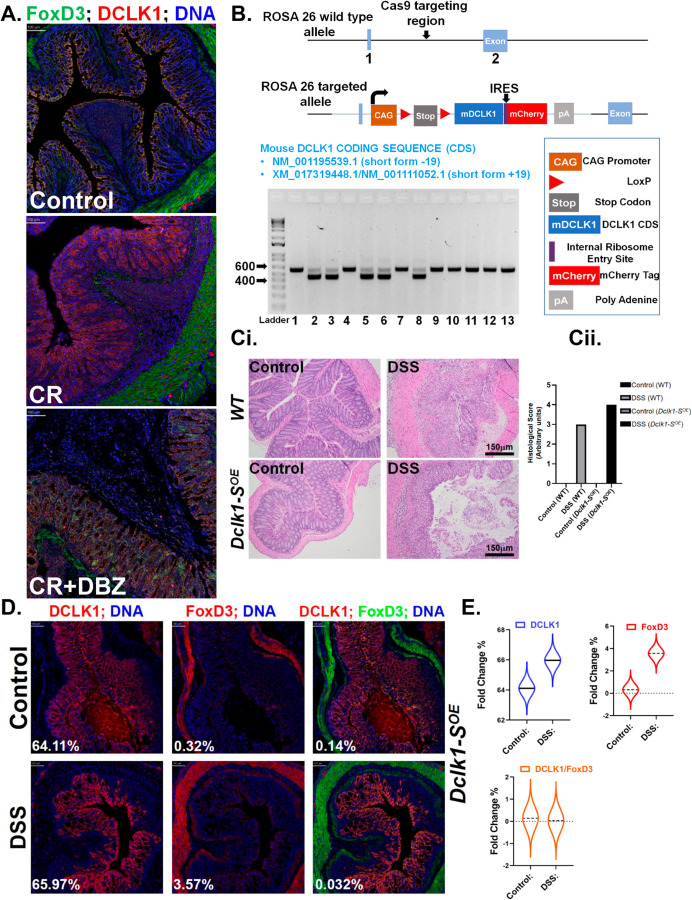
DCLK1-S persists during colon inflammation and displays an inverse correlation with FOXD3. A. Using the IMC data sets from Dclk1^ΔIEC^ mice, DCLK1 (red) is overlayed with FoxD3 (green) in the indicated groups. B. Development of DCLK1-S^OE^ mice. Mouse genomic fragments containing homology arms (HAs) were amplified using high fidelity Taq and were subsequently assembled into a targeting vector together with recombination sites and selection markers shown. *Dclk1*-Short+19-floxed mice were bred with *Villin*-Cre mice to generate *Dclk1*-Short+19-floxed; *Villin*-Cre ${\mathrm{DCLK1-S}}^{\text{OE}}$ mice. Mice were subsequently genotyped. Ci. WT littermates and ${\mathrm{DCLK1-S}}^{\text{OE}}$ mice received 4% DSS in drinking water for 7 days and euthanized after 2 days of normal water. Representative H&E staining (n = 6 mice/genotype) to visualize crypt architecture. Scale bar = 150 *μ*m. Cii. Bar graph showing histological scores. D. IMC showing DCLK1(red) staining overlayed with DNA (blue), FoxD3 (red) staining overlayed with DNA (blue) and DCLK1 (red)/FoxD3 (green) and DNA (blue) colocalization in the Control or DSS-treated ${\mathrm{DCLK1-S}}^{\text{OE}}$ mice. Scale bars as indicated; n = 6 mice/genotype. E. Percent fold change based on staining intensity in DCLK1+ cells, fold change of DCLK1 is in blue, fold change of FoxD3 is in red, and the ratio of both DCLK1/FoxD3 is in orange.

Thus, despite lack of an antibody specifically dedicated for the detection of DCLK1-S, we infer from these findings that CR-induced loss of DCLK1-S repressor FoxD3 could potentially help in the upregulation of DCLK1-S in our model. To further substantiate this notion, we overlayed the DCLK1 and FoxD3 staining in the same sections and observed that the two proteins did not co-localize in the CR group (Fig 2A). With more severe colitis in the CR+DBZ group, we observed a rebound in FoxD3 staining concomitant with a decline in DCLK1 further indicating an inverse relationship (Fig 2A). This was further confirmed via western blotting (S3A Fig).

Encouraged by these findings, we next developed a knock-in mouse model over-expressing DCLK1-S using exon 19 (DCLK1-S +19) under the CAG promoter. Fig 2B provides the schematic of the knock-in strategy and genotypes after breeding them with mice expressing constitutive *Villin-Cre*. When control *(fl/fl)* or *floxed; Villin-Cre (fl/+;*${\text{Dclk1-S}}^{\text{OE}}$) mice were subjected to 4% DSS in drinking water for 7 days, we discovered severe histopathological changes, including extensive crypt loss and pronounced ulceration in the ${\text{Dclk1-S}}^{\text{OE}}$ mice compared to *fl/fl* mice (Fig 2Ci and 2Cii). To ensure DCLK1-S overexpression in the colons of these mice, we next subjected sections prepared from control or DSS-treated ${\text{Dclk1-S}}^{\text{OE}}$ mice to IMC with antibodies against DCLK1 and FoxD3. Fig 2D clearly depicts DCLK1-S overexpression in DSS-treated colons compared to control. At the same time, FoxD3 staining or DCLK1-FoxD3 co-stainings were barely detectable consistent with FoxD3 being a negative regulator of DCLK1-S (Fig 2D). Moreover, given previous indications of DCLK1’s potential regulation of the Notch pathway [23,29], we examined the levels of Notch intracellular domain (NICD) in our +19 mouse group. Interestingly, we observed a significant increase in NICD levels within the DSS-treated groups (S3B Fig). These findings suggest a sustained expression of DCLK1-S, which correlates inversely with FoxD3 and positively with NICD expression during inflammation.

### DCLK1-S expression during inflammation coincides with elevated levels of MMP13 expression and activity

We examined the mRNA fold changes for selected inflammatory genes in addition to DCLK1-S and MMP13 in colon explants from WT mice normalized to GAPDH control (S4A Fig). The data show an elevated expression of both DCLK1-S and MMP13 in response to CR infection, followed by a moderate decrease in their expression in CR+DBZ-treated samples (S4A Fig). Additionally, we used IMC to check for the levels of MMP13 in tissue sections of our DCLK1^ΔIEC^ model of CR-induced colitis. These data showed an elevated level of MMP13 particularly in the crypt and stromal region of CR infected mice with more localized staining in the stromal region with more severe colitis (S4B-S4D Fig).

**Fig 3:**
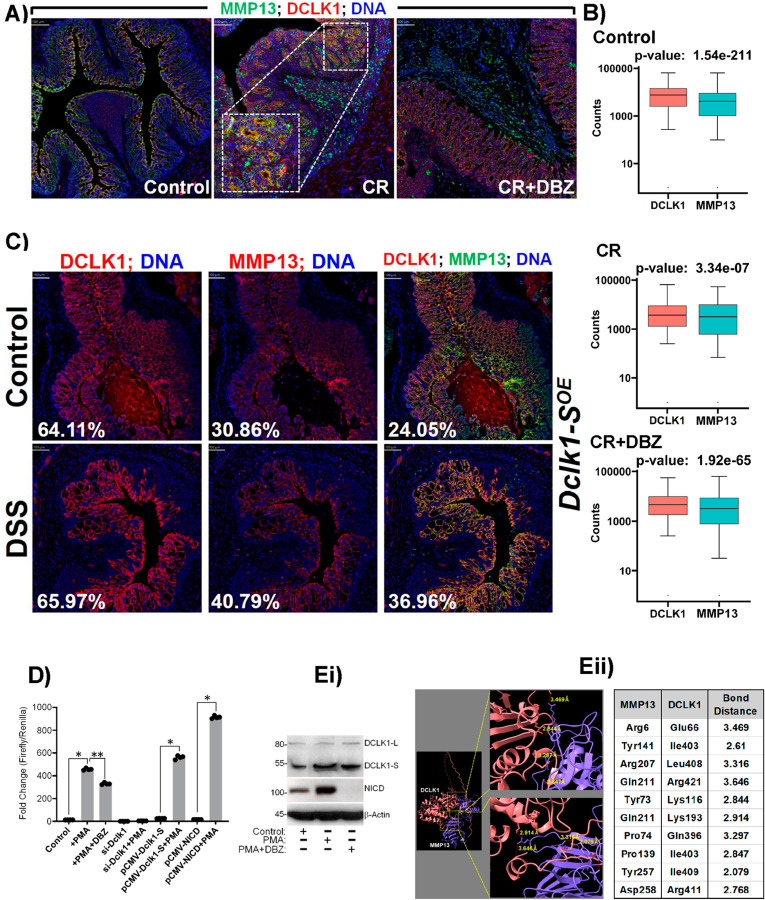
Co-localization of MMP13 with DCLK1 in various models of colitis. A. Paraffin sections prepared from the colons of Control, CR or CR+DBZ group of Dclk1^ΔIEC^ mice were subjected to IMC. MMP13 (green) is overlayed with DCLK1 (red) and DNA (blue). Boxed areas in CR group indicates significant co-localization of MMP13 with DCLK1. Scale bars as indicated (100 *μ*m); 8-10 mice /group. B. Box plots of DCLK1 and MMP13 counts based on IMC data set in the Control, CR, CR+DBZ groups. C. DCLK1 and MMP13 staining from IMC in control and *Dclk1-S*^OE^ group after DSS-induced colitis. Lane 1 DCLK1(red) staining is overlayed with DNA (blue), Lane 2 MMP13 (red) staining is overlayed with DNA (blue), Lane 3 DCLK1(red)/MMP13(green)/DNA(Blue) colocalization in the *Dclk1-S*^OE^ mice. P values as indicated. D. MMP13 promoter-reporter activity (*, **p<0.05; n = 3 independent experiments). Ei. Western blot data of HCT116 colon cancer cells treated with PMA and PMA+DBZ. Eii. In silico molecular docking studies to predict MMP13 and DCLK1 binding. CR: *Citrobacter rodentium*, CR+DBZ: *Citrobacter rodentium* + Dibenzazepine (DBZ), PMA: Phorbol 12-Myristate 13-Acetate.

We sought to further explore the relationship between DCLK1-S and MMP13. Colocalizing the DCLK1 staining with MMP13 from our IMC analysis, we observed minimal staining of both proteins in the uninfected controls. In response to CR infection however, levels of DCLK1 increased in tandem with MMP13, with both proteins colocalizing in the crypt epithelial compartment (Fig 3A).

In the CR+DBZ group, significant expression of both genes persisted, but DCLK1 remained predominant in the crypt, while MMP13 was more prevalent in the stromal area (Fig 3A). Analyzing the cell count in the separate groups based on our IMC data, we noted a higher number of DCLK1-positive cells compared to MMP13 in the control groups. However, in the CR group, the numbers became approximately equal, and MMP13-positive cell counts declined in the CR+DBZ group (Fig 3B). Additionally, by examining the staining patterns of DCLK1 and MMP13 in the colons of ${\text{Dclk1-S}}^{\text{OE}}$ mice, we observed a similar pattern of colocalization. DCLK1 minimally colocalized with MMP13 in the untreated controls, but this colocalization became more prominent following DSS-induced colitis (Fig 3C).

Furthermore, we wanted to clearly ascertain the molecular interactions that exist between DCLK1-S and MMP13. We cloned MMP13 promoter and performed the promoter-reporter activity in HEK-293 cells. Fig 3D revealed increased promoter activity in response to Phorbol myristate acetate (PMA) with partial decline in PMA+DBZ-treated cells. To determine if DCLK1-S is playing any role in MMP13 activity, cells knocked down for DCLK1 with siRNA, failed to respond to PMA (Fig 3D). Ectopic overexpression of DCLK1-S on the other hand, improved the baseline activity (Fig 3D). PMA treatment of cells overexpressing DCLK1-S however, exhibited a dramatic increase in promoter activity compared to PMA alone, suggesting that DCLK1-S may influence MMP13 function. To see if Notch signaling regulates MMP13 activity, we next transiently transfected HEK-293 cells harboring the MMP13 promoter with a Notch intracellular domain (NICD)-containing plasmid (pCMV-NICD) and treated cells with PMA which led to a significant increase in MMP13 promoter activity (Fig 3D). To rule out the possibility that DCLK1-L isoform may have a similar role, we treated HCT116 colon cancer cells with either PMA or PMA+DBZ and examined the levels of DCLK1-L and -S isoforms along with NICD. Fig 3Ei shows that DCLK1-L levels were not affected by either treatment. DCLK1-S isoform however was induced by PMA and partially decreased in response to PMA+DBZ. As expected, Notch signaling was significantly inhibited in PMA+DBZ-treated cells compared to PMA alone (Fig 3Ei). Thus, both DCLK1-S and Notch signaling seem critical in influencing MMP13 function.

To examine if DCLK1 directly interacts with MMP13, we used AlphaFold, the deep learning algorithm developed by DeepMind [30]. We discovered significant accuracy in predicting the three-dimensional structure of the two proteins as the interaction between the two proteins was strong, based on the binding distance (Fig 3Eii).

**Fig 4:**
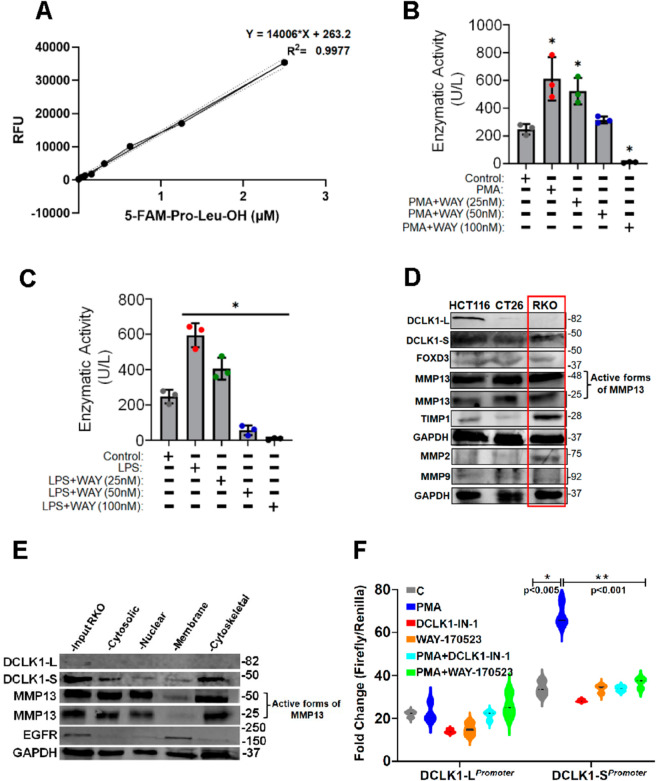
MMP13 enzyme activity, subcellular distribution, DCLK1-L and DCLK1-S promoter-reporter assays. RKO cells were treated with PMA or PMA plus selective and potent MMP13 inhibitor WAY 170523 at varying doses as indicated for 30 min followed by measurement of enzymatic activity. A. Reference curve showing relative fluorescence units (RFUs). B, C. Dose-dependent decrease in MMP13 enzymatic activity (n = 3 independent experiments; *p<0.05). D. Western blots showing relative protein abundance in three colon cancer cell lines. Boxed area represents levels of the indicated proteins in RKO cells. E. Subcellular compartmentalization of proteins from RKO cells. 1: Cytosolic Fraction, 2: Nuclear Fraction, 3: Membrane Fraction, 4: Cytoskeletal fraction (n = 3 independent experiments). F. Promoters for DCLK1-L and DCLK1-S isoforms were cloned and transfected in HEK293 cells and promoter-reporter activity assays were performed using Dual-Luciferase Reporter Assay System (E1910, Promega, Madison, WI). Luminescence was measured using a BioTek Synergy Neo luminometer. P values as indicated; n = 3 independent experiments.

Before exploring the effect of DCLK1-MMP13 interaction on MMP13 function, we standardized MMP13 activity assay using SensoLyte 520 MMP-13 Fluorimetric Assay Kit (Cat.# AS-72019; AnaSpec, Freemont, CA). In Fig 4A, the 5-FAM-Pro-Leu-OH reference standard is depicted, serving as a reference for MMP13 enzymatic activity in units per liter (U/L).

By utilizing RKO colon cancer cell line, we discovered that in response to PMA, a significant increase in MMP13 enzymatic activity was recorded with a dose-dependent decrease in response to MMP13 inhibitor WAY-170523 (Cat.# 2633, TOCRIS Biosciences; Fig 4B). We repeated the same experiment with Lipopolysaccharide (LPS), a structural component of Gram-negative bacteria. Treatment with LPS resulted in a similar increase in MMP13 enzyme activity with a dose-dependent decrease in response to WAY-170523 (Fig 4C). During western blot of MMP13 in various colon cancer cell lines, active forms of MMP13 could be seen in all three (HCT116, RKO, CT-26) cell lines along with DCLK1-S while FoxD3 levels were minimally detected (Fig 4D). Likewise, the levels of two other MMPs, MMP2 and MMP9 were fewer than MMP13 (Fig 4D). Since tissue inhibitor of metalloproteinase-1 (TIMP-1) negatively regulates MMP13, we also probed cells with antibody for TIMP1. As shown in Fig 4D, TIMP1 levels were relatively higher in RKO cells when compared with the other two cell lines but still fewer than MMP13 suggesting a complex regulatory mechanism. Next, we fractionated RKO cells into membrane, cytosolic, nuclear, and cytoskeletal compartments and probed with various antibodies. Fig 4E shows presence of MMP13 in cytosolic, nuclear, and cytoskeletal but not membrane fractions. This correlated with presence of DCLK1-S in the cytosolic and more so in the cytoskeletal fraction (Fig 4E). Since the cytoskeleton can affect extracellular remodeling in a number of ways including EMT [31], it is plausible that cytoskeletal DCLK1-S interacts with MMP13 to influence extracellular matrix remodeling.

To further connect DCLK1 isoforms with MMP13, we cloned promoters for both DCLK1-L and DCLK1-S isoforms and performed promoter-reporter activities employing MMP13 inhibitor WAY-170523 and DCLK1 inhibitor, DCLK1-IN-1, respectively. Following transfection into HEK293, cells were treated with PMA and WAY-170523 or DCLK1-IN-1 either separately or in combination with PMA. Treatment with PMA led to a subtle increase in DCLK1-L promoter activity (Fig 4F). In response to either WAY-170523 or DCLK1-IN-1, we observed a decrease in promoter activity which did not reach significance (Fig 4F). While combining DCLK1-IN-1 with PMA did not reverse inhibition, PMA+WAY-170523 rescued DCLK1-L promoter activity (Fig 4F). When DCLK1-S promoter activities were measured, significant increases in promoter activity in response to PMA were irreversibly inhibited by WAY-170523 or DCLK1-IN-1 added separately or in combination with PMA (Fig 4F) suggesting that DCLK1-S’ interaction with MMP13 may be more integral to MMP13’s role as an inflammatory mediator.

**Fig 5:**
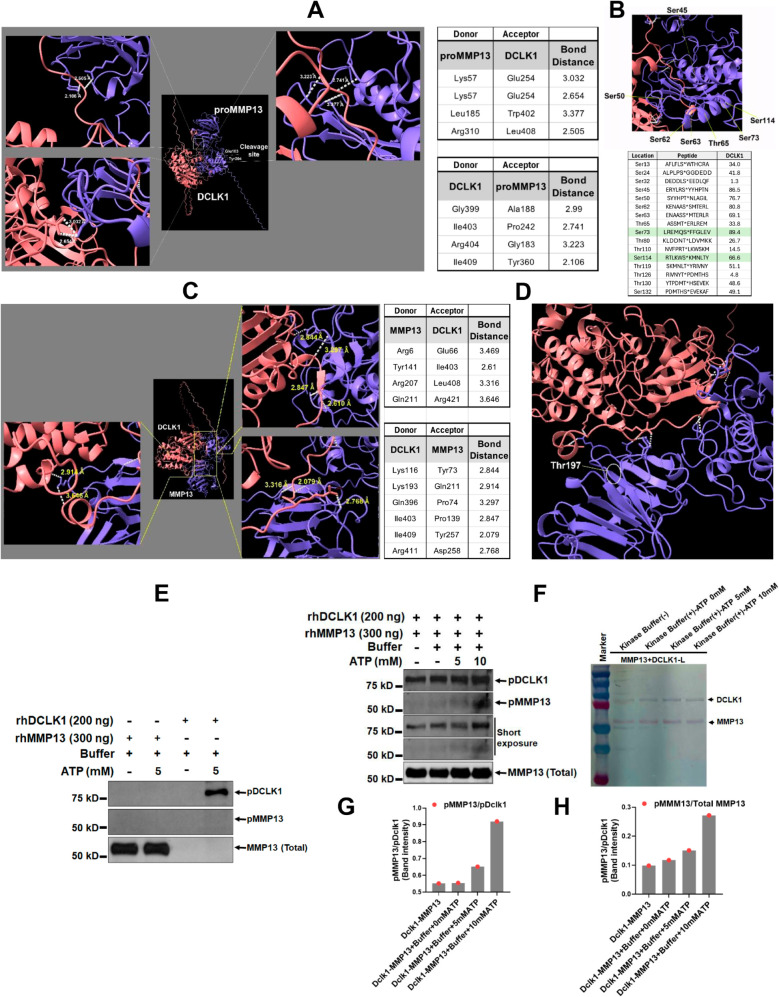
Molecular docking and in vitro kinase assays. A-D. Molecular docking study showing the interaction of proMMP13 (A) and mature MMP13 (C) with DCLK1-S. B. Ser 73 and Ser 114 highlighted in green in proMMP13, represent the phosphorylation sites for DCLK1-S. D. Arg207 and Gln211 are donor sites from MMP13 in mature protein close to Thr197 (circled). E. Both rhMMP13 and rhDCLK1 were incubated in kinase buffer in presence or absence of ATP at 30 °C for 1 hr. rhMMP13 didn’t show any autophosphorylation in the same condition. Phosphorylation was detected by pan phospho-Serine/Threonine antibody. F. rhMMP13 was incubated with rhDCLK1 in presence of ATP at 30 °C for 1 hr. Phosphorylated rhMMP13 was detected with immunoblotting using pan phospho-Serine/Threonine antibody. Representative immunoblots showing the levels of phosphorylation of rhMMP13. Right panel represents the Coomassie staining (n = 3 independent experiments). G, H. Protein band intensity ratios, pMMP13/pDCLK1 and pMMP13/Total MMP13, showing rhMMP13 phosphorylation by rhDCLK1.

To deep dive into the mechanism(s) through which DCLK1-S may be impacting MMP13’s function, *We hypothesized that DCLK1-S (short form +19) may be interacting with pro-MMP13 and may be integral to its activation through phosphorylation.* As depicted in Fig 5A, following molecular docking, we discovered that both MMP13 and DCLK1 can be H donors. Interestingly, there was interaction between DCLK1 and proMMP13 around the cleavage site of proMMP13 (Glu103). In proMMP13, it was Lys57, Leu185 and Gly183. Next, to see if there are Ser and Thr around these sites that may be potential sites for phosphorylation, we used Phosphositeplus site based on the method developed by Lewis Cantley [32,33].

Fig 5B shows Ser 73 and Ser114 in pro-MMP13 and the peptide that was used to predict percentile phosphorylation for DCLK1 and DCLK2. These are within the region where interaction takes place as well as cleavage of proMMP13. Next, following up from Fig 3Eii wherein the docking revealed that DCLK1 can also interact with mature MMP13, we discovered that while DCLK1-S interacts with both pro-MMP13 and cleaved MMP13, there were clear differences. Only Arg310 (pro-MMP13): Leu408 (DCLK1) and Arg207 (MMP13) and Leu408 (DCLK1) were common to both (Fig 5A and 5C). Interestingly, Thr197 in mature MMP13 appears to be a target for DCLK1-S as Arg207 and Gln211 are donor sites from MMP13, very close to Thr197 (Fig 5D). While detailed biochemical assays confirming these interactions are ongoing, we performed in vitro kinase assays with rhDCLK1 and rhMMP13 proteins and discovered that while DCLK1 was autophosphorylated consistent with previous report [34], MMP13 did not show autophosphorylation under similar assay conditions (Fig 5E). When rhMMP13 was incubated with rhDCLK1 in presence of ATP at 30 °C for 1 hr, rhDCLK1 dose-dependently phosphorylated rhMMP13 as was detected with immunoblotting using pan phospho-Serine/Threonine antibody (Fig 5F). Since the assay buffer from Cell Signaling Technology (https://www.cellsignal.com) contains Glycerophosphate, it is not surprising that DCLK1 exhibited baseline phosphorylation (Fig 5F). We therefore measured band intensities in the presence or absence of 5 and 10mM ATP and as shown in Fig 5G and 5H, we did notice augmentation of MMP13 phosphorylation. Thus, it appears that DCLK1 could directly phosphorylate MMP13; yet more studies are needed to clearly understand how each isoform of DCLK1 regulates MMP13 function via phosphorylation.

### MMP13, upregulated in both *Dclk1*^ΔIEC^ and *Dclk1-S*^OE^ mouse colons, co-localizes with ECM proteins and provides a plausible mechanism for EMT and fibrosis

**Fig 6:**
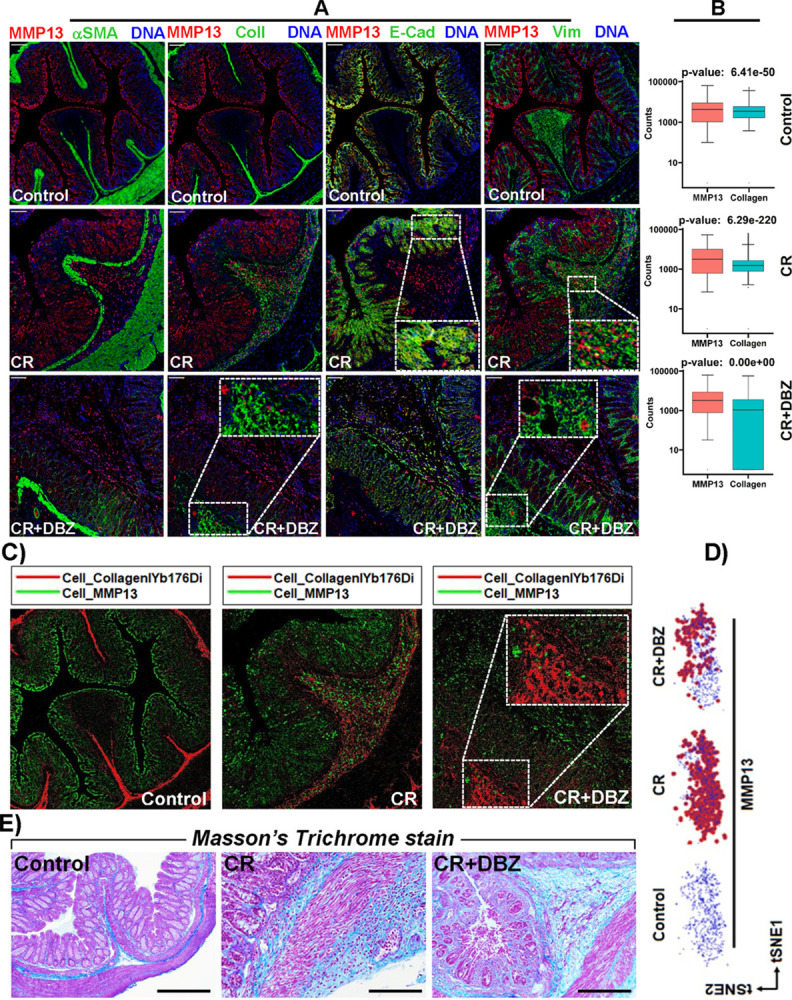
MMP13 upregulation coincides with changes in ECM, EMT and fibrosis markers in vivo in Dclk1^ΔIEC^ mice. A. Tissue sections prepared from the colons of indicated groups of mice were subjected to immunostaining with antibodies against MMP13 (red), *α*-SMA (green), Collagen (green), E-cadherin (green) and Vimentin (green). Samples were analyzed using the Hyperion Imaging System (Standard BioTools). DAPI (blue) was used to label DNA. Scale bars = 100 *μ*m (n = 8-10mice/group). B. Box plots of MMP13 and Collagen counts based on IMC data set in the Control, CR and CR+DBZ groups. C. An overlay of Collagen (Red) and MMP13 (green) in tissue sections of the indicated groups. The boxed area represents a magnified image of collagen accumulation in the CR+DBZ group. Scale bars = 100 *μ*m (n = 8-10mice/group). D. t-SNE plots showing MMP13 intensity across different groups. E. Masson’s Trichrome staining of tissue sections prepared from the colons of Control, CR, and CR+DBZ mice. Please note increases in collagenous fibrous tissue (stained blue) in both CR and CR+DBZ groups. Scale bars = 200 *μ*m; n = 8-10mice/group. CR: *Citrobacter rodentium*, CR+DBZ: *Citrobacter rodentium* + Dibenzazepine (DBZ).

MMPs are involved in both augmenting and attenuating many processes that impact fibrosis. To further understand the impact of DCLK1 interaction with MMP13 on MMP13’s function, our next step was to assess how MMP13 interacts with both epithelial and extracellular matrix proteins including E-Cadherin, *α*-SMA, Collagen, and Vimentin. In the colons of *Dclk1*^ΔIEC^ mice, we observed an increase in MMP13 staining in CR-infected colons compared to control wherein, MMP13 co-localized with E-cadherin in the epithelial region while MMP13 co-localization was also seen between Collagen I and Vimentin but not with *α*-smooth muscle actin (*α*-SMA) in the sub- epithelial regions (Fig 6A-C).

In the CR+DBZ group, we observed a partial decrease in MMP13 staining leading to accumulation of Collagen I in the sub-epithelial region suggesting ECM remodeling (Fig 6A-C, inset). Two-dimensional t-SNE plots confirmed increases in MMP13 in the CR group with a partial decline in the CR+DBZ group (Fig 6D). Fig 6E represents Masson’s Trichrome staining showing collagenous fibers in both CR and CR+DBZ group.

To connect DCLK1 and its spatial distribution relative to other extracellular matrix (ECM) proteins, we investigated the co-localization of DCLK1 and FoxD3 with protein markers *α*-SMA, E-Cadherin, Collagen, and Vimentin (S5A and S5B Fig). In the uninfected control groups, we observed minimal staining of DCLK1, with no colocalization with *α*-SMA, Collagen, and Vimentin, but some overlap with E-cadherin staining at the crypt edges (S5A Fig). Additionally, a few DCLK1+ cells were detected in the stromal area (S5A Fig). FoxD3 staining also showed minimal expression in uninfected controls, with a distinct staining pattern observed compared to DCLK1 (S5B Fig). During inflammation in the CR group, there was an expansion of DCLK1+ cells throughout the crypt, accompanied by increased collagen expression in the stromal area, elevated E-cadherin positive cells colocalizing with DCLK1, and increased vimentin expression spreading across both stromal and epithelial compartments (S5A Fig). In this group, FoxD3 expansion was observed in separate, distinct compartments of the colon that colocalized with *α*-SMA but not with Collagen, E-cadherin, or Vimentin (S5B Fig). In the CR+DBZ group, denoting a more severe form of colitis, aberrant crypt architecture correlated with a decline in DCLK1 staining (S5A Fig). Collagen deposition persisted in the stromal area of the colon. Compared to the E-cadherin and Vimentin staining in CR, the CR+DBZ group showed fewer E-cadherin/DCLK1 localizations but more robust DCLK1/Vimentin colocalization (S5A Fig). In the CR+DBZ group, FoxD3 staining also expanded into the crypt area and showed some colocalization with *α*-SMA, E-cadherin, and Vimentin (S5B Fig). The observed trends indicate an upregulation of extracellular matrix (ECM) remodeling proteins, including MMP13, collagen, and vimentin, which may initially be activated as part of a wound healing response to inflammation. However, these proteins become sustained as inflammation persists and promote fibrosis.

### DCLK1-S driven inflammation alters the colon cells to display an EMT-like phenotype

**Fig 7:**
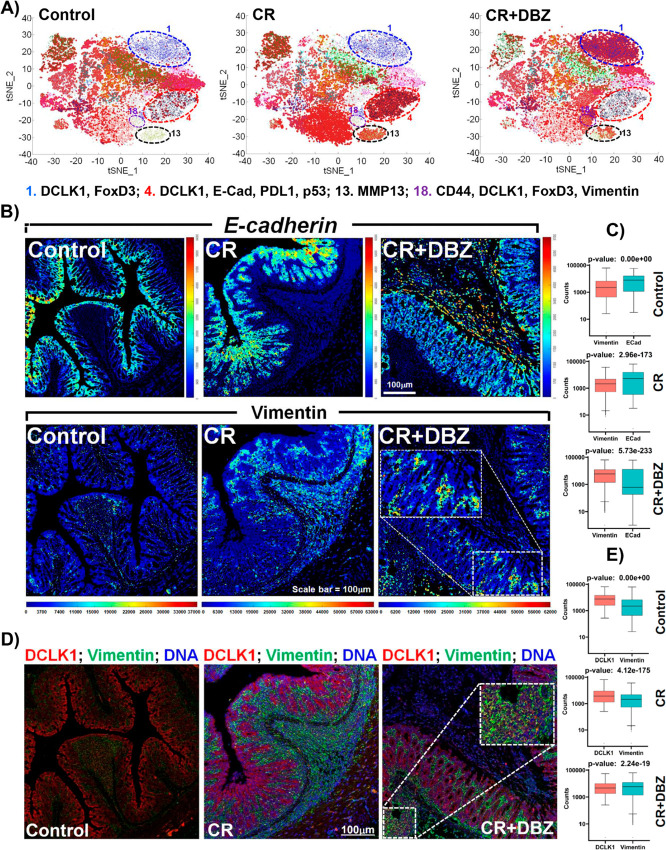
Loss of E-cadherin and increased deposition of Vimentin suggests an EMT phenotype with DCLK1-S driven inflammation in the colons of Dclk1^ΔIEC^ mice. A. Two-dimensional t-SNE plots showing the clusters and relative abundances of cells. Numbers below represent markers associated with the indicated clusters. B. Representative heatmap channels generated for E-cadherin and Vimentin in the indicated groups using MCD-Viewer. Boxed area in CR+DBZ group represents higher Vimentin intensity. Scale bars as indicated; n = 8-10mice/group. C. Box plots of Vimentin and E-cadherin counts are based on the IMC data set in the Control, CR and CR+DBZ groups. P values as indicated. D. DCLK1(red) Vimentin (green) and DNA (blue) staining in the indicated groups. Boxed area represents DCLK1-Vimentin co-localization. Scale bars as indicated; n = 8-10mice/group. E. Box plots of DCLK1 and Vimentin counts based on IMC data set in the Control, CR, CR+DBZ groups. CR: *Citrobacter rodentium*, CR+DBZ: *Citrobacter rodentium* + Dibenzazepine (DBZ).

Based on the distinct colon clustering algorithm, staining observed across different groups (S2C Fig), we conducted an analysis of the cellular phenotype of colon cells present during inflammation using tSNE plots and phenograph from the IMC datasets (S2C and S2D Fig). Twenty-one clusters were identified based on all the markers utilized, with some clusters overlapping the different specific proteins of interest (S2C and S2D Fig). For the purposes of this study, we focused on examining the clusters associated with DCLK1, MMP13, FoxD3, Vimentin, and E-cadherin (Fig 7A)

DCLK1 expression was associated with clusters 1, 4, and 18, MMP13 with cluster 13, FoxD3 with clusters 1 and 18, Vimentin with cluster 18, and E-cadherin with cluster 4, respectively (Figs 7A, S2C and S2D). Consistent with previous observations, cluster 1 is a cluster of DCLK1 and FoxD3. The data presented reveals an abundant DCLK1 expression present at the onset of inflammation but was overcome by an abundance of FoxD3 in the CR+DBZ group (Fig 7A). Mirroring the pattern observed for DCLK1, MMP13 expression in cluster 13 shows an increase during inflammation but was decreased in the CR+DBZ group (Fig 7A). Interestingly, there was a shift between E-cadherin and Vimentin levels as inflammation progressed. E-cadherin levels appeared to increase in the CR group but decreased as inflammation proceeded, while Vimentin levels intensified with more severe colitis (Fig 7A, 7B and 7C). Co-localization of DCLK1 staining with Vimentin revealed a rapid increase in Vimentin expression levels as inflammation progressed and was sustained with more severe colitis (Fig 7D), which is in clear contrast to the E-cadherin/DCLK1 patterns previously observed. When comparing DCLK1 IMC counts to Vimentin counts in the cells at different groups, it was evident that Vimentin counts were lower in the Control and CR group but became elevated with the CR+DBZ group (Fig 7E). These results imply that when DCLK1-S induced inflammation progresses, there is a transition from an epithelial to a more mesenchymal phenotype in the colon to potentially facilitate EMT.

## Discussion

Inflammatory bowel diseases present significant challenges in terms of management and treatment due to their chronic and progressive nature. Although existing treatment protocols aim to ameliorate inflammation, they often come with a host of adverse effects. Ulcerative colitis specifically targets the colon, causing persistent inflammation that can lead to various complications. While the introduction of advanced therapeutics such as biologic drugs and small compounds has revolutionized UC treatment, remission rates and treatment durability remain modest [35,36]. Given the complexity and severity of UC, there is a compelling need to delve deeper into the molecular mechanisms underlying colitis to identify novel therapeutic targets and improve patient outcomes.

Expanding upon prior investigations emphasizing the importance of DCLK1 and its isoform in gastrointestinal function, this study investigates the role of the DCLK1 isoform (DCLK1-S) in colitis and modulation of inflammation. Leveraging *Dclk1*^fl/fl^ mice, which enable tamoxifen-induced deletion of the DCX domains of DCLK1, we aimed to discern the specific impact of DCLK1-S in this context. Utilizing the CR model of infectious colitis, which mirrors human colitis symptoms [37,38], we embarked on the first investigation probing the specific role of DCLK1-S in this model using high-throughput Imaging Mass Cytometry. IMC, an innovative technique merging mass cytometry (CyTOF) with in situ immunohistochemistry (IHC), allows for the concurrent detection of over 40 biomarkers in tissue sections using stable metal isotope-conjugated antibodies or genomic probes. By leveraging specialized algorithms, IMC extracts single-cell data, including marker expression and spatial coordinates, to provide a comprehensive platform for tissue exploration, discovery, and in-depth analysis of tissue structure. This method enables detailed examination of cell numbers, localization, phenotypes, and network interactions, providing valuable insights into biological processes within tissues [26,27,39]. Our findings reveal that persistent expression of DCLK1-S fosters inflammation, ECM remodeling via MMP13 upregulation, fibrosis, and epithelial-to-mesenchymal transition. These processes are hallmark features of IBD progression, underscoring the pivotal role of DCLK1-S persistence in regulating these processes.

Our observations in both *Dclk1*^ΔIEC^ and *Dclk1-S*^OE^ mouse models revealed that inflammation led to a sustained expression of DCLK1-S, which exhibited a negative correlation with FoxD3 expression. Conversely, as colitis severity increased in the CR+DBZ group, there was a slight decrease in DCLK1 expression accompanied by an increase in FoxD3 status. These findings align with prior research indicating the inflammatory-promoting role of DCLK1-S [12,19,23] and the negative regulatory function of FoxD3 on DCLK1-S [28]. Given FoxD3’s frequent methylation in colon cancers [40], it is plausible that the increased expression of DCLK1-S in tissues is due to the downregulation of FoxD3 status in colitis. The decrease in epithelial DCLK1 upon the addition of the Notch inhibitor DBZ mirrors previous research indicating that heightened DCLK1-S status aligns with Notch activity, a correlation reaffirmed by our study.

Studies have shown that DCLK1-L acts as a gatekeeper, maintaining homeostasis under stress, while DCLK1-S becomes pathogenic when DCLK1-L is absent or suppressed. Indeed, DCLK1-S activates oncogenic signaling, enhances stemness, and promotes epithelial-mesenchymal transition [11,29,41]. Thus, loss of DCLK1-L may create a permissive environment, but the presence and persistence of DCLK1-S may determine whether inflammation resolves or progresses to neoplasia. We can glean from these studies that loss of DCLK1-L primes the mucosa, but additional inflammatory signals may be required to fully drive transformation. We propose that loss of DCLK1-L is a key initiating event, but not solely sufficient to cause colitis or colon cancer. Instead, disease progression likely results from a convergence of DCLK1-L loss, DCLK1-S upregulation, and environmental stressors. Whether triggered by epithelial loss of DCLK1-L, immune dysregulation, or direct overexpression, the presence of DCLK1-S consistently correlates with epithelial plasticity, impaired resolution, and inflammation-fueled transformation.

During the course of investigation, we further noted that the upregulation of MMP13 coincided with significant co-staining with DCLK1-S in the colons of both *Dclk1*^ΔIEC^ and *Dclk1-S*^OE^ mice. While previous studies have elucidated the role of MMPs in the pathogenesis of IBD and other inflammatory conditions [42,43], this marks the first instance of associating DCLK1-S-driven inflammation with MMP13 activity, although MMP13 has previously been implicated in IBD progression [44,45]. The decline in MMP13 in the CR+DBZ group coincided with increased collagen deposition and colon tissue fibrosis associated with inflammation. Additionally, we noted the transition of epithelial cells to a more mesenchymal state, as evidenced by the upregulation of extracellular matrix components along with Vimentin, coupled with a reduction in epithelial-positive cells. This epithelial-to-mesenchymal transition (EMT) phenotype aligns with previous studies that have underscored the role of DCLK1 in promoting EMT in various cancers [41,46,47]. These findings highlight a novel and significant role of DCLK1-S in promoting colitis progression by facilitating extracellular matrix accumulation, fibrosis, and EMT, highlighting novel pathways that can be targeted in the course of colitis treatment.

To this end, MMP13 is known to function as an ECM-degrading enzyme in osteoarthritis joints [48,49]. MMP13 cleaves multiple collagens as well as other extracellular matrix (ECM) substrates such as gelatin, fibronectin, and aggrecan relevant to tumor metastasis [50]. Increased expression of MMP13 has also been associated with poor prognosis in patients with colorectal cancer metastasis to the liver [51]. Likewise, IBD-related inflammation is associated with increased expression of MMPs [44], and studies in DSS model of colitis specifically reported exaggerated expression of MMP-3 and MMP-9 [51]. During subcellular distribution, we discovered that both DCLK1-S and MMP13 were detected in the cytoskeletal fractions thereby implying that DCLK1-S-MMP13 axes is likely to expose the cryptic domains within ECM molecules to trigger both neutrophil infiltration and EMT as a prelude to colitis. As a corollary to this, Neutrophil Elastase (NE), a proteoglycan-degrading enzyme, has recently been shown to activate pro-MMP13 [52]. In prior publication [23], we have shown that Ly6G+ neutrophils express very high levels of DCLK1-S. While this mode of regulation of MMP13 by NE remains to be determined, our in silico molecular docking study revealed that DCLK1-S interacts with pro-MMP13 around the cleavage site at Glu103 with Ser73 and Ser114 serving as the potential phosphorylation sites for DCLK1-S indicating that post-translational modification at these sites probably promotes conversion of pro-MMP13 into active MMP13. We also discovered that DCLK1-S interacts with mature MMP13 with Thr197 acting as a potential phosphorylation site for DCLK1-S. Multiple mechanisms of MMP-13 activation exists [53], including epigenetic modification [54–56], transcriptional or post-transcriptional regulation by ncRNAs, and the activation or inhibition of proenzymes [48,49]. We glean from our findings that DCLK1-S’ interaction with pro-MMP13 probably causes activation of the latent form of the protein while it’s interaction with mature MMP13 may be required for MMP13’s role as a modifier of ECM remodeling. Our in vitro kinase assays provide a probable mechanism of MMP13 regulation by direct phosphorylation by DCLK1. However, several other mechanisms could be in play in the regulation of MMP13 function in our models, including CR-induced activation of NF-κB [57,58] that regulates DCLK1-S [14]. Indeed, recent studies have suggested that during inflammation, DCLK1 kinase can phosphorylate IKK*β* [59–61], presenting the possibility that inflammation-driven upregulation of DCLK1-S serves as an initiating event in DCLK1-S-mediated inflammation. This process may be perpetuated by the continuous phosphorylation of IKK*β* by DCLK1-S, creating a self-sustaining cycle of chronic inflammation. Finally, Notch-induced Hes1 release is shown to regulate MMP13 [62]. Thus, more studies are needed to fully comprehend DCLK1 isoform-specific regulation of MMP13-driven inflammation and colitis.

## Conclusion

Ulcerative Colitis, a prevalent form of IBD lacking a definitive cure, poses significant challenges for treatment. To explore novel avenues for colitis management, we employed IMC in a mouse model of infectious colitis encompassing prevalent DCLK1-S expression. Our investigations unveiled a correlation between DCLK1-S upregulation and MMP13 activity, accompanied by increased expression of ECM remodeling proteins and EMT factors. These findings shed light on several molecular mechanisms underlying IBD progression, offering insights into potential targets for treatment discovery.

## Materials and methods

**Ethics Statement**. This investigation adhered strictly to the guidelines outlined in the National Institutes of Health’s Guide for the Care and Use of Laboratory Animals. Approval for all methods employed was obtained from the Animal Care and Use Committee at the University of Kansas Medical Center (ACUP Number: 23-10-353).

### Mouse models and colitis induction

To develop colitis in age-and sex-matched littermates or wild type (WT) controls and *Dclk1*^ΔIEC^mice (8 mice/group) [23], CR was administered via drinking water or oral gavage for 16-24 hours following an overnight culture of CR (biotype 4280, ATCC, 10^8^ colony-forming units), which was identified as pink colonies on MacConkey agar, in accordance with established protocols [23,63]. To inhibit Notch signaling, mice were intraperitoneally administered a cell-permeable *γ*-secretase inhibitor, DBZ (EMD Chemicals, Inc.), at a dose of 10 *μ*mol/kg of body weight suspended in 0.5% (wt/vol) hydroxypropyl-methylcellulose and 0.1% Tween-80 in water (wt/vol.) [64] on postinfection days 4 and 6. The mouse *Dclk1* gene has 20 exons that undergo alternative splicing to produce multiple isoforms [65]. We developed a knock-in mouse model over-expressing DCLK1-S, using exon 19 (DCLK1-S +19) under the CAG promoter. Following breeding with mice expressing constitutive *Villin-Cre*, littermate control or *Dclk1-S*^OE^ mice were genotyped using the following primers: F1: 5’-CACTTGCTCTCCCAAAGTCGCTC-3’; R1: 5’-ATACTCCGAGGCGGATCACAA-3’; R2: 5’-AGATGTACTGCCAAGTAGGAAAGTC-3’. Eight weeks old control or *Dclk1-S*^OE^ mice (8 mice/group) received 4% DSS in drinking water for 7 days and switched to normal water for 2 days before euthanasia and tissue collection.

### Imaging mass cytometry workflow

Colon tissue samples from the control, CR infected or CR+DBZ-treated *Dclk1*^ΔIEC^ and control or DSS-treated *Dclk1-S*^OE^ mouse groups were fixed in formalin. Following fixation, the tissues were embedded either in paraffin or frozen. Paraffin-embedded tissue sections were prepared and mounted on slides. These slides were then sent to Fluidigm, now referred to as Standard BioTools Inc., for downstream imaging mass cytometry (IMC) analysis. Specific antibodies targeting proteins of interest were utilized for the IMC, with catalog numbers provided in S2 Fig A. These antibodies were conjugated with heavy metal isotopes using commercially available kits or custom conjugation services. The tissue sections were deparaffinized and rehydrated, with antigen retrieval performed if necessary. Subsequently, the sections were incubated with the metal-conjugated antibodies to allow binding to their target antigens. Once stained, the tissue sections were loaded onto specialized slides compatible with the IMC instrument. These slides were introduced into the IMC instrument, which underwent laser ablation scanning. As the laser ablated the tissue, metal ions released from the metal-conjugated antibodies were detected by a mass spectrometer. On 3 regions of interest (ROIs), Fluidigm performed single cell segmentation based on the IMC Cell Segmentation kit and provided excel reports with single-cell level intensity and coordinate values for every cell and each marker to facilitate further quantitative assessment and spatial mapping. Fluidigm also provided single-cell analysis consisting of tSNEs, heatmaps, and phenographs for each of the 3 ROIs. This process generated spatially resolved data on the expression levels of the target proteins across the tissue section. The raw IMC data obtained was processed using specialized software such as MCD Viewer or histoCAT. This allowed for visualization and analysis of the spatial distribution of proteins within the tissue [66].

### Masson’s trichrome staining

Fixation, paraffin embedding, and thin sectioning of tissue sections from Control, CR, and CR+DBZ were performed. Weigert’s iron hematoxylin, Biebrich scarlet-acid fuchsin, phosphomolybdic-phosphotungstic acid, and aniline blue solutions are used to stain sections after they have been deparaffinized and rehydrated. Following staining, slices are cleaned, dried, mounted, and inspected with inverted microscope to assess the staining features of the nucleus, collagen fibers, and muscle fibers.

### Crypt and crypt-denuded lamina propria (CLP) isolation for RT-PCR

Crypts containing epithelial cells were isolated from distal colons according to established protocols. Following crypt removal, CLPs were extracted, and both isolated crypts and CLPs underwent further processing for biochemical and molecular assays [25]. Total RNA extraction from the isolated crypt epithelial cells was performed using Trizol (Invitrogen). Subsequently, reverse transcription was carried out to convert RNA into complementary DNA (cDNA) utilizing a High-Capacity cDNA Reverse Transcription Kit (Thermo Fisher Scientific). Quantitative real-time polymerase chain reaction (qRT-PCR) was then conducted to assess mRNA levels of the target genes. Primers specific to the genes of interest, as listed in S1 Table, were utilized and GAPDH was used as the housekeeping gene. The qRT-PCR reaction mixture (Thermo Fisher) comprised cDNA template, gene-specific primers, and SYBR Green dye. The qRT-PCR reactions were conducted on a real-time PCR instrument (Thermo Fisher). The threshold cycle (Ct) values obtained from the qRT-PCR, utilizing the comparative Ct method $2^{-\Delta \Delta C_{t}}$ were employed to determine the relative mRNA expression levels.

### Transfection and luciferase assays

HEK 293T cells were plated at $6.0\;\times \;10^{4}$ cells/cm^2^ in 24- well plates (353047, Corning, NY). Cells were transfected in each well using 1.5 *μ*l Lipofectamine 3000 (L3000008, Invitrogen, Carlsbad, CA), 1.5 *μ*l P3000 reagent, ∼100 ng of hRL-TK transfection efficiency internal control construct, and 1.5 *μ*g of MMP13, DCLK1-L or DCLK1-S promoter luciferase constructs. After ∼24 hr of post-transfection, drug treatment was performed for 24 hrs. Cells were lysed in 1× lysis buffer (E1531, Promega, Madison, WI) and analyzed for luciferase activity using Dual-Luciferase Reporter Assay System (E1910, Promega, Madison, WI). Luminescence was measured using a BioTek Synergy Neo luminometer.

### MMP13 enzyme activity measurement

MMP13 activity assay was performed using the MMP-13 Fluorimetric Assay Kit (Cat.# AS-72019; AnaSpec, Fremont, CA). Enzyme activity was determined using a fluorescently labelled peptide (FRET peptide) as the substrate, according to the manufacturer’s instructions. Briefly, 50 *μ*l of MMP13 substrate solution (100 *μ*M) was mixed with 50 *μ*l of normalized sample in a black 96-well microtiter plate. The fluorescence signal (expressed as relative fluorescence units) was monitored at Ex/Em = 490 nm/520 nm upon MMP13-induced cleavage of the FRET peptide using a luminescence spectrometer (PerkinElmer LS55).

### In silico MMP13/DCLK1 docking

We used AlphaFold, the deep learning algorithm developed by DeepMind [30], to model both pro-MMP13 and mature MMP13 in complex with the DCLK1 Short Form (SF), +19 variant. For collagenase-3 preproprotein [Homo sapiens], the NCBI Reference Sequence NP_002418.1 >NP_002418.1 collagenase 3 preproprotein [*Homo sapiens]* was used: MHPGVLAAFLFLSWTHCRALPLPSGGDEDDLSEEDLQFAERYLRSYYHPTNLAGILKENAASS MTERLREMQSFFGLEVTGKLDDNTLDVMKKPRCGVPDVG E **YNV**FPRTLKWSKMNLTYRIVNY TPDMTHSEVEKAFKKAFKVWSDVTPLNFTRLHDGIADIMISFGIKEHGDFYPFDGPSGLLAHAF PPGPNYGGDAHFDDDETWTSSSKGYNLFLVAAHEFGHSLGLDHSKDPGALMFPIYTYTGKSHF MLPDDDVQGIQSLYGPGDEDPNPKHPKTPDKCDPSLSLDAITSLRGETMIFKDRFFWRLHPQQ VDAELFLTKSFWPELPNRIDAAYEHPSHDLIFIFRGRKFWALNGYDILEGYPKKISELGLPKEVKK ISAAVHFEDTGKTLLFSGNQVWRYDDTNHIMDKDYPRLIEEDFPGIGDKVDAVYEKNGYIYFFN GPIQFEYSIWSNRIVRVMPANSILWC. The first three amino acids of the cleaved protein **YNV** are highlighted. Cleavage occurs after the Glutamate at position 103 (E underlined above). To identify Ser and Thr around the cleavage sites, we used phosphositeplus based on the method developed by Lewis Cantley [32,33].

### In vitro DCLK1 kinase activity assay

To examine DCLK1 binding/kinase activity, we used recombinant human DCLK1 protein (full length, rhDCLK1 (D14-10H, Sino Biologicals) and recombinant human MMP13 protein, rhMMP13 Pro form (511-MM-010, R&D Systems). In brief, around 200 ng rhDCLK1 and 300 ng rhMMP13 were mixed with 1X Kinase buffer (25 mM Tris-HCl, pH 7.5, 5 mM b-Glycerophosphate, 2 mM DTT, 0.1 mM Na_3_VO_4_, 10 mM MgCl_2_; Cell Signaling Technology: www.cellsignal.com) in a 25 mL reaction volume containing ATPs. Samples were incubated at 30°C for 1 hr and stopped by 4× SDS sample loading buffer, followed by 5 min heating at 98°C. Phosphorylated proteins were detected by immunoblotting using pan Phospho-Serine/Threonine Rabbit mAb (AP1475, ABclonal, MA) and mouse mAb (sc-515284, Santa Cruz) for total MMP13.

### Western blot analysis

HCT116, RKO, and CT-26 cells were cultured and harvested for protein extraction using RIPA buffer. Protein concentration was determined, and 50 *μ*g of protein was denatured by heating with SDS and *β*-mercaptoethanol. Gel electrophoresis was performed to separate proteins by size. The separated proteins were transferred onto a membrane, which was subsequently blocked to prevent nonspecific binding. The membrane was incubated with primary antibodies against DCLK1 (1:500, ab31704), FoxD3 (1:500, PA5-106539), MMP2 (1:500, NB200-114SS), MMP9 (1:500, sc-393859), MMP13 (1:500, sc-515284), EGFR (1:1000, CST 4267S), NICD/Notch1 (1:500, CST #3447S), GAPDH (1:1000, sc-32233), TIMP1 (1:500, MA1-773), *β*-actin (1:10,000, A3854), and *β*-tubulin (1:1000, D3U1W). Secondary antibodies, either goat anti-mouse or anti-rabbit IgG, were used for detection.

For subcellular compartmentalization of proteins, the Compartment Protein Extraction Kit (EMD Millipore, Cat#2145) was used to isolate different cellular compartments. Subsequently, 40 *μ*g of protein from each subcellular fraction was analyzed via western blotting as described above.

### Statistical analysis

Values are expressed as mean ± standard deviation (SD) or standard error of the mean (SEM) for histology scores. For two-group comparisons, statistical analyses were performed using an unpaired, two-sided Student’s *t*-test. For comparisons involving more than two groups, one-way analysis of variance (ANOVA) was conducted, followed by Fisher’s protected least significant difference (PLSD) post hoc test. Statistical significance was defined as *p* < 0.05. All analyses were performed using GraphPad Prism, version 9.

## Supporting information

S1 Fig Imaging mass cytometry (IMC) reveals distinct cellular compartments in the colons of Dclk1^ΔIEC^ mice. A. DNA overlay in tissue sections from the indicated groups. B. Cell overlay in tissue sections of the indicated groups. C. Tissue sections from the indicated groups stained with antibodies against E-cadherin (red) and *α*-SMA (green) were analyzed using the Hyperion Imaging System (Standard BioTools). D. Bright-field images of E-cadherin and *α*-SMA staining in tissues from the indicated groups. Scale bars = 200 *μ*m; *n* = 2 independent experiments. CR, *Citrobacter rodentium*; CR+DBZ, *Citrobacter rodentium* + Dibenzazepine (DBZ).
(TIF)

S2 Fig A. Panel and antibody design used for imaging mass cytometry (IMC) studies. B. Phenograph overlay of all markers assessed in the study, with 21 clusters identified in the colons of *Dclk1*^ΔIEC^ mice. C. Heatmaps and tSNE color guide for all identified clusters, showing marker expression intensity within each cluster. D. tSNE plots depicting cellular changes observed in each group, representing the 21 identified clusters.
(TIF)

S3 Fig A. Western blot showing accumulation of FoxD3 in CR+DBZ-treated crypt extracts from the colons of Dclk1^ΔIEC^ mice. B. DSS-induced colitis promotes increased Notch signaling. Tissue sections prepared from the colons of Control or DSS-treated *Dclk1-S*^OE^ mice were stained with antibodies against DCLK1 and NICD. DAPI was used to label DNA. Significant co-staining between DCLK1-S and NICD was observed in DSS samples (*n* = 3 independent experiments).
(TIF)

S4 Fig DCLK1 expression correlates with MMP13 levels A. Expression profile of indicated markers in colon explants from WT mice. Partial inhibition of DCLK1-S and MMP13 expression is observed in the CR+DBZ group (boxed areas). *p* values as indicated; experiments run in triplicate. B, C. Bright-field and immunofluorescence images of MMP13 staining in sections prepared from the colons of *Dclk1*^ΔIEC^ mice from IMC. Scale bars = 100 *μ*m; *n* = 2 independent experiments. D. Representative heatmap channels generated for MMP13 in the indicated groups using MCD-Viewer. Scale bars = 100 *μ*m; *n* = 2 independent experiments.
(TIF)

S5 Fig FoxD3 negatively regulates DCLK1, and sustained expression of DCLK1 promotes extracellular matrix (ECM) remodeling and epithelial-mesenchymal transition (EMT) in Dclk1^ΔIEC^ mice. A. DCLK1 (red) is overlaid with *α*-SMA, collagen, E-cadherin, and vimentin.B. FoxD3 (red) is overlaid with *α*-SMA, collagen, E-cadherin, and vimentin. Scale bars as indicated; *n* = 2 independent experiments.
(TIF)

S1 Table List of primers used for rt-PCR. (PDF)

S1 File Raw data files for IMC data sets. (ZIP)
