# Supplementary figures and images for: DCLK1 isoform (DCLK1-S) as a critical player in promoting inflammation, tissue remodeling, and EMT in mouse models of colitis
Source: PLoS Pathog. 2025 Aug 21;21(8):e1013360. doi: 10.1371/journal.ppat.1013360 (PMC12370143; doi:10.1371/journal.ppat.1013360)

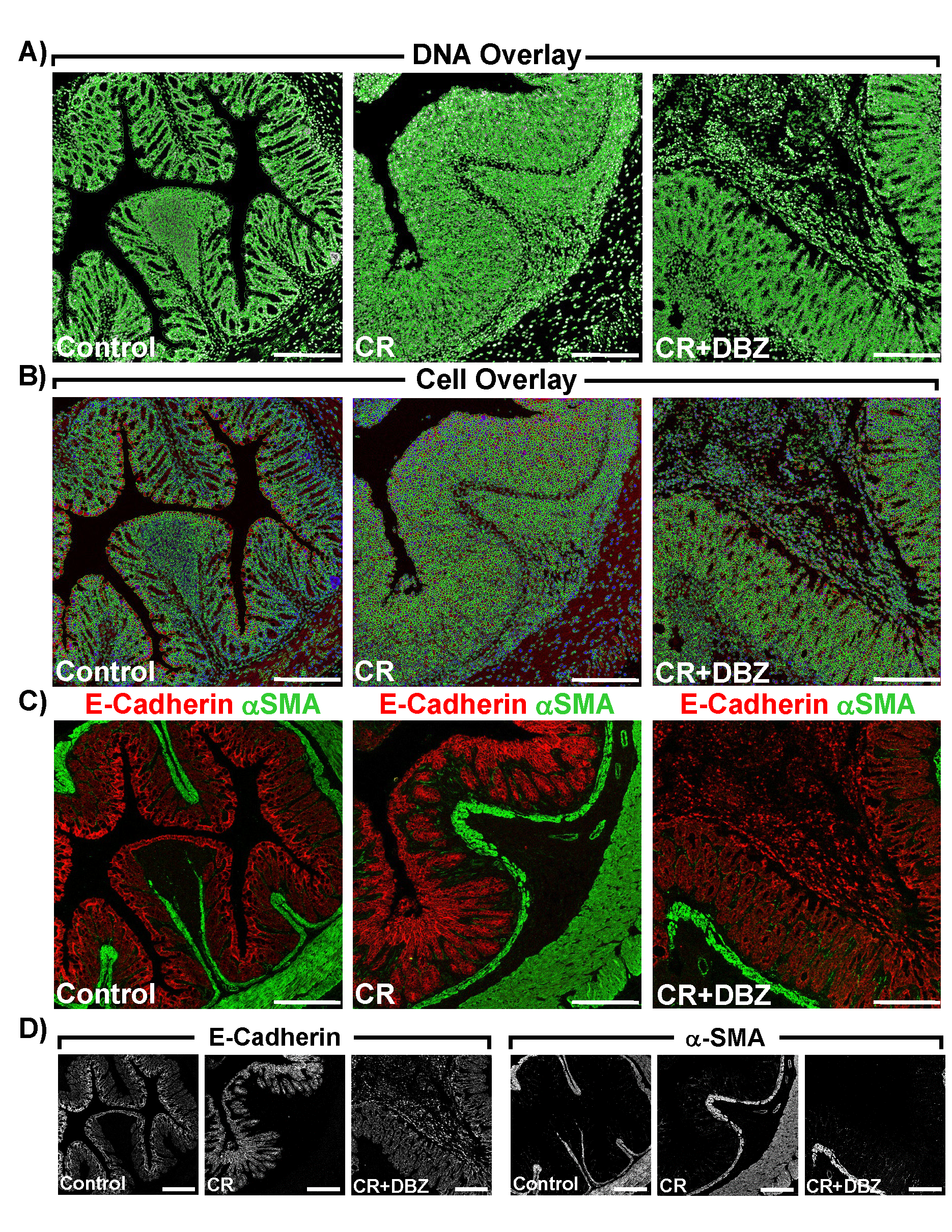

Supplement: S1 Fig — A. DNA overlay in tissue sections from the indicated groups. B. Cell overlay in tissue sections of the indicated groups. C. Tissue sections from the indicated groups stained with antibodies against E-cadherin (red) and α-SMA (green) were analyzed using the Hyperion Imaging System (Standard BioTools). D. Bright-field images of E-cadherin and α-SMA staining in tissues from the indicated groups. Scale bars = 200 μm; n = 2 independent experiments. CR, Citrobacter rodentium; CR+DBZ, Citrobacter rodentium + Dibenzazepine (DBZ). (TIF) [file ppat.1013360.s001.tif]

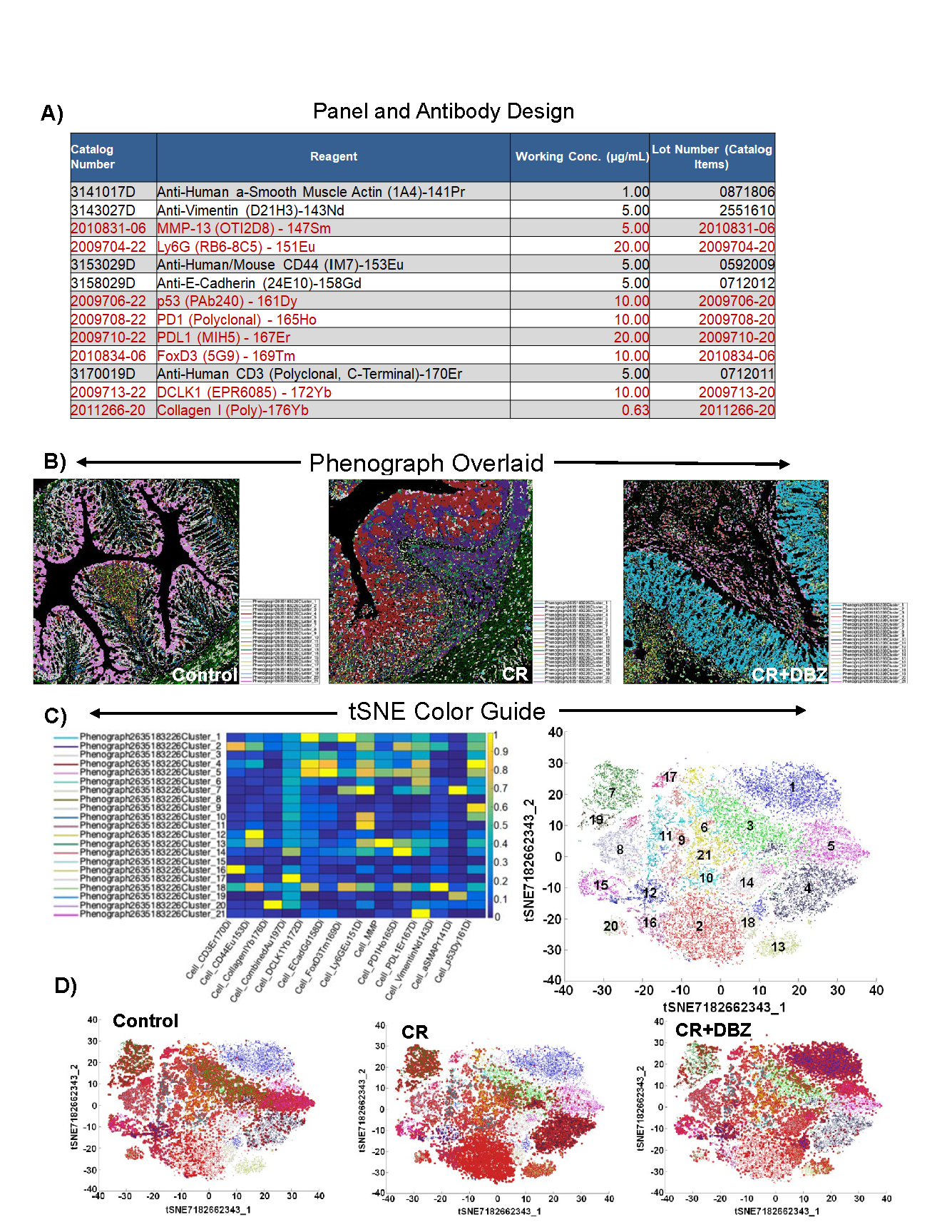

Supplement: S2 Fig — B. Phenograph overlay of all markers assessed in the study, with 21 clusters identified in the colons of Dclk1ΔIEC mice. C. Heatmaps and tSNE color guide for all identified clusters, showing marker expression intensity within each cluster. D. tSNE plots depicting cellular changes observed in each group, representing the 21 identified clusters. (TIF) [file ppat.1013360.s002.tif]

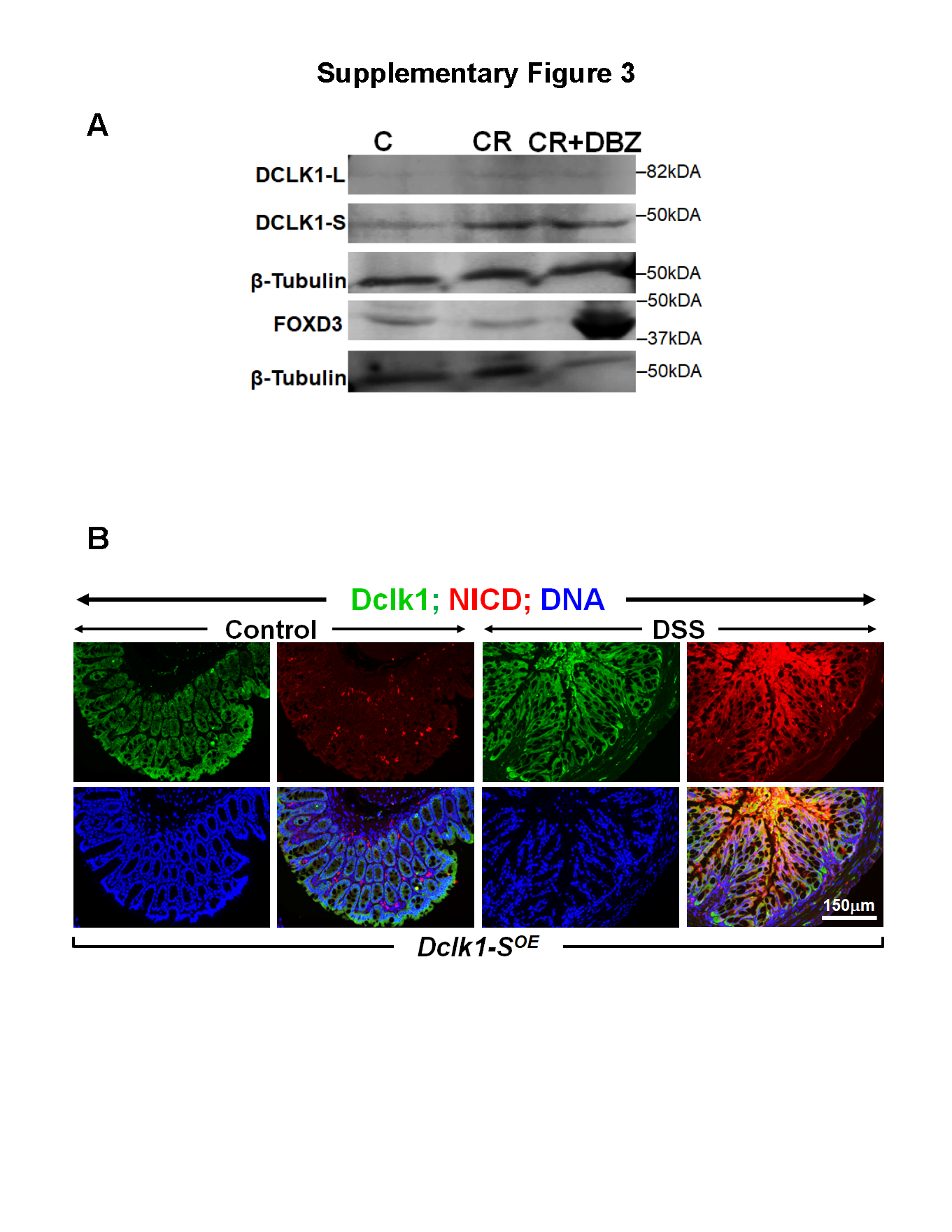

Supplement: S3 Fig — B. DSS-induced colitis promotes increased Notch signaling. Tissue sections prepared from the colons of Control or DSS-treated Dclk1-SOE mice were stained with antibodies against DCLK1 and NICD. DAPI was used to label DNA. Significant co-staining between DCLK1-S and NICD was observed in DSS samples (n = 3 independent experiments). (TIF) [file ppat.1013360.s003.tif]

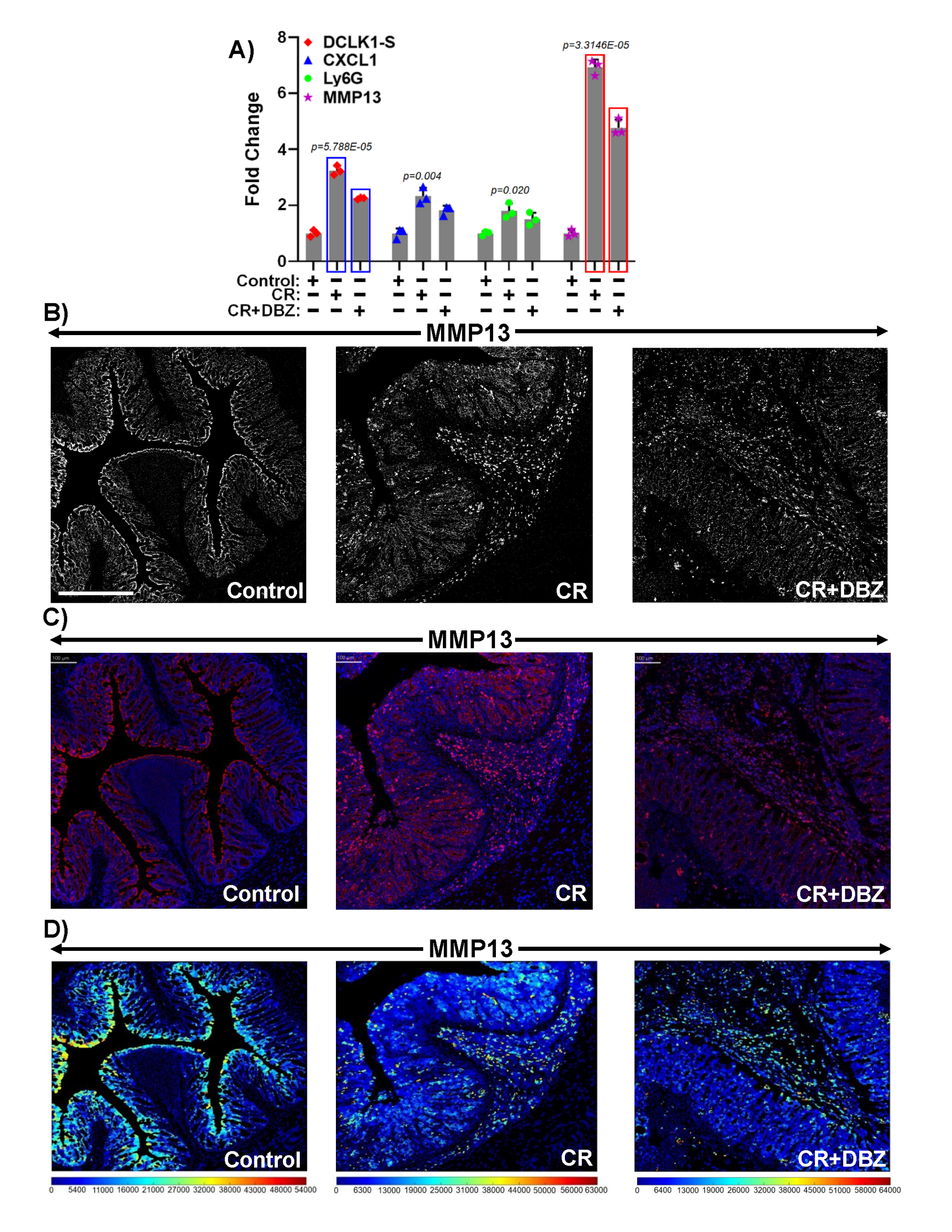

Supplement: S4 Fig — A. Expression profile of indicated markers in colon explants from WT mice. Partial inhibition of DCLK1-S and MMP13 expression is observed in the CR+DBZ group (boxed areas). p values as indicated; experiments run in triplicate. B, C. Bright-field and immunofluorescence images of MMP13 staining in sections prepared from the colons of Dclk1ΔIEC mice from IMC. Scale bars = 100 μm; n = 2 independent experiments. D. Representative heatmap channels generated for MMP13 in the indicated groups using MCD-Viewer. Scale bars = 100 μm; n = 2 independent experiments. (TIF) [file ppat.1013360.s004.tif]

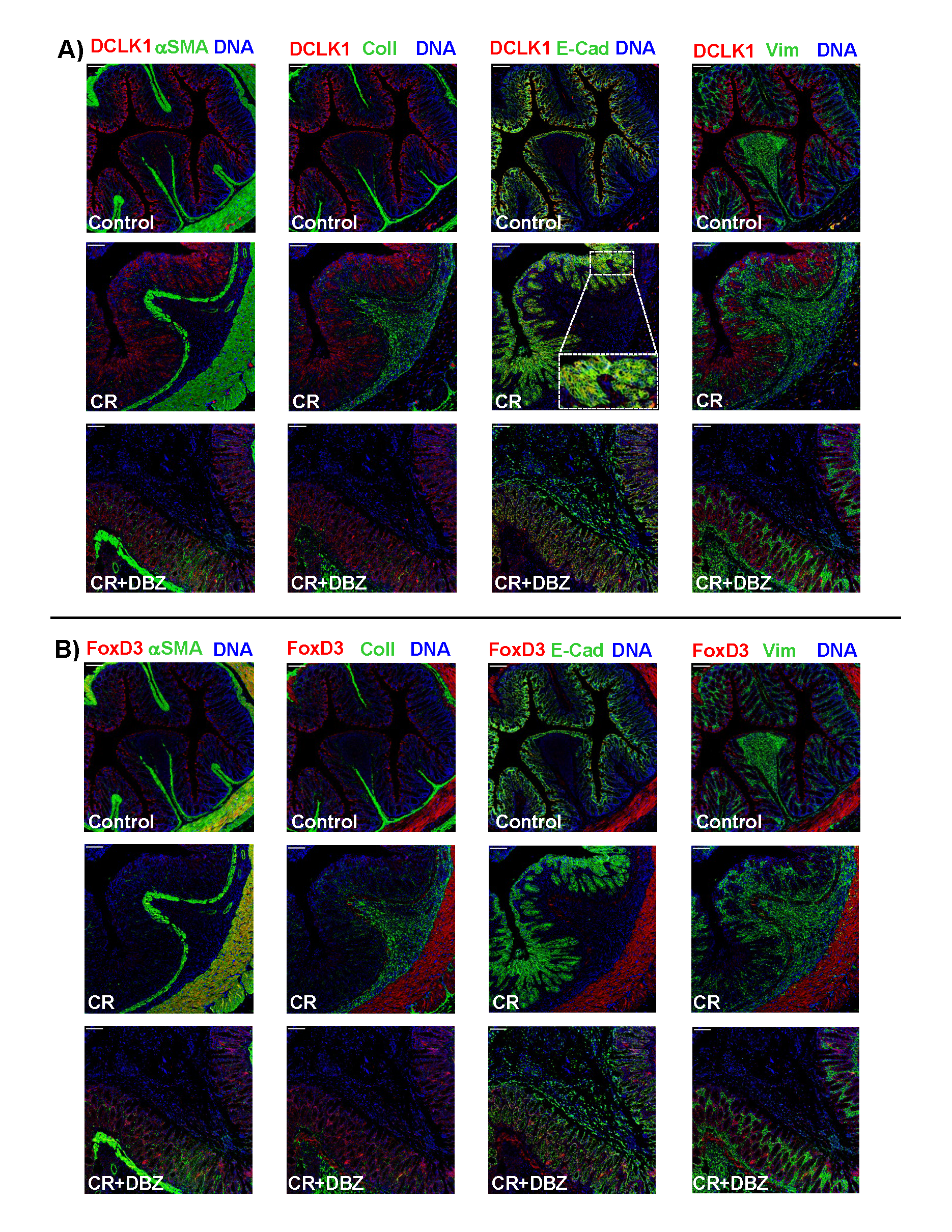

Supplement: S5 Fig — A. DCLK1 (red) is overlaid with α-SMA, collagen, E-cadherin, and vimentin.B. FoxD3 (red) is overlaid with α-SMA, collagen, E-cadherin, and vimentin. Scale bars as indicated; n = 2 independent experiments. (TIF) [file ppat.1013360.s005.tif]
