# Supplementary material for: DCLK1 isoform (DCLK1-S) as a critical player in promoting inflammation, tissue remodeling, and EMT in mouse models of colitis
Source: PLoS Pathog. 2025 Aug 21;21(8):e1013360. doi: 10.1371/journal.ppat.1013360 (PMC12370143; doi:10.1371/journal.ppat.1013360)
Supplement: S1 Table — (PDF) [file ppat.1013360.s006.pdf]

Supplementary Table 1: Primers for rt-PCR

Genes	Forward Primers	Reverse Primers
Mouse GAPDH	aactttggcattgtggaagg	acacattgggggtaggaaca
Mouse Dclk1-S	gtcagccttacgcaggaaaa	tgggaagcagttggattagc
Mouse Cxcl-1	cttgaaggtgttgccctcag	tggggacaccttttagcatc
Mouse Ly6G	ttgcaaagtcctgtgtgctc	aggggcaggtagttgtgttg
Mouse MMP13	gagccacagatgagcacaga	atgtaaggccacctccactg
